# A comprehensive fungi-specific 18S rRNA gene sequence primer toolkit suited for diverse research issues and sequencing platforms

**DOI:** 10.1186/s12866-018-1331-4

**Published:** 2018-11-20

**Authors:** Stefanos Banos, Guillaume Lentendu, Anna Kopf, Tesfaye Wubet, Frank Oliver Glöckner, Marlis Reich

**Affiliations:** 1 Molecular Ecology, Institute of Ecology, FB02, University of Bremen, Leobener Str. 2, 28359 Bremen, Germany; 2 Department of Soil Ecology, Helmholtz Centre for Environmental Research GmbH – UFZ, Halle-Saale, Germany; 3 Department of Ecology, University of Kaiserslautern, Kaiserslautern, Germany; 4 Microbial Genomics and Bioinformatics Research Group, Max Planck Institute for Marine Microbiology, Bremen, Germany; 5 Present address: Department of Community Ecology, Helmholtz Centre for Environmental Research GmbH – UFZ, Halle-Saale, Germany; 6 Department of Life Sciences and Chemistry, Jacobs University Bremen gGmbH, Bremen, Germany; 7 German Centre for Integrative Biodiversity Research (iDiv) Halle-Jena-Leipzig, Leipzig, Germany

**Keywords:** Fungi, 18S rRNA gene sequence (SSU) primer, Annealing blocking oligonucleotides, Co-amplification, Real-time Q-PCR, Fungal biodiversity, Taxonomic classification, Community survey, FR-1, FF390

## Abstract

**Background:**

Several fungi-specific primers target the 18S rRNA gene sequence, one of the prominent markers for fungal classification. The design of most primers goes back to the last decades. Since then, the number of sequences in public databases increased leading to the discovery of new fungal groups and changes in fungal taxonomy. However, no reevaluation of primers was carried out and relevant information on most primers is missing. With this study, we aimed to develop an 18S rRNA gene sequence primer toolkit allowing an easy selection of the best primer pair appropriate for different sequencing platforms, research aims (biodiversity assessment versus isolate classification) and target groups.

**Results:**

We performed an intensive literature research, reshuffled existing primers into new pairs, designed new Illumina-primers, and annealing blocking oligonucleotides. A final number of 439 primer pairs were subjected to in silico PCRs. Best primer pairs were selected and experimentally tested. The most promising primer pair with a small amplicon size, nu-SSU-1333-5′/nu-SSU-1647-3′ (FF390/FR-1), was successful in describing fungal communities by Illumina sequencing. Results were confirmed by a simultaneous metagenomics and eukaryote-specific primer approach. Co-amplification occurred in all sample types but was effectively reduced by blocking oligonucleotides.

**Conclusions:**

The compiled data revealed the presence of an enormous diversity of fungal 18S rRNA gene primer pairs in terms of fungal coverage, phylum spectrum and co-amplification. Therefore, the primer pair has to be carefully selected to fulfill the requirements of the individual research projects. The presented primer toolkit offers comprehensive lists of 164 primers, 439 primer combinations, 4 blocking oligonucleotides, and top primer pairs holding all relevant information including primer’s characteristics and performance to facilitate primer pair selection.

**Electronic supplementary material:**

The online version of this article (10.1186/s12866-018-1331-4) contains supplementary material, which is available to authorized users.

## Background

Fungi belong to a highly diverse kingdom providing key ecosystem functions. Additionally, their biosynthesis of natural products relevant for biotechnological application renders them of great interest to the research community. Yet, they are a highly understudied group with an estimated species number of up to 3.8 million but only about 120,000 being described [1]. Thus, detection and accurate classification represents one of the critical bottlenecks for fungal research.

Molecular taxon identification is mainly based on marker gene sequencing whose sensitivity, resolution and throughput are controlled by the choice of the marker gene and sequencing platform. While Sanger-sequencing is the standard for single taxon identification, Illumina MiSeq and to a less extent third generation sequencing techniques are the bases for community surveys. Fungal marker genes differ in length, resolution power among different fungal groups, phylogenetic power, number of publicly available sequences and available suitable primer sets [2]. The Internal Transcribed Spacer (ITS) region is the proposed barcode for fungi as it has species resolution for a very broad range of fungi compared to other fungal marker genes [3]. However, many fungal taxa recovered by environmental ITS-sequencing can often be identified solely to kingdom or phylum level due to the lack of reference sequences or reference sequences annotated only to high taxonomic levels [4]. One solution is the use of a phylogenetic marker beside the ITS allowing a phylogeny-based assignment of the fungal sequences. Hereby, sequences are inserted into a fungal phylogenetic reference tree to transfer the taxonomic information of the given branch on the query [5]. Thus, sequences originating from unknown fungal taxa can often be assigned to a lower taxonomic level. Such a double-marker gene approach has been shown to be effective in surveys targeting communities mainly composed by undescribed fungal taxa [6, 7]. In the case that the aim of a research project is the analysis of the structure and dynamic of fungal communities rather than the monitoring of known fungal taxon groups, phylogenetic marker sequencing is a promising approach.

Similarly, single taxon identification often depends on multiple markers for a precise classification to a lower taxonomic level. The first step is often a phylogeny-based classification with a pre-marker gene guiding further steps for a taxonomic fine-tuning with a group-specific marker [8].

The most prominent fungal phylogenetic markers are the 28S and the 18S rRNA gene sequences [9]. Though the 28S rRNA gene often resolves to a lower taxonomic level, most of the publicly available sequence data are 18S rRNA gene sequences [10]. In the last decades, several 18S rRNA gene sequence primers have been designed as fungi-specific, however, characteristics, overall fungal and group-specific coverage rate, and possible co-amplification with non-fungal eukaryotic taxa are rarely reported [11, 12] and comparisons among primer pairs are generally lacking.

The presented primer toolkit aims to systematically simplify the choice of the correct primer pair dependent on the research aim (community survey versus isolate classification), sequencing platform, and fungal target group. The analysis included an intensive literature research, compilation of primer, primer and annealing blocking oligonucleotides design, followed by in silico and empirical evaluation of the primer performance. The outcome is a toolkit comprising of most comprehensive lists of primers (pairs) reporting characteristics, referenced annealing position, coverage of variable regions, overall and subphyla-specific coverage rate, and co-amplification rate for a total of 164 primer, 439 primer pairs and four annealing blocking oligonucleotides.

## Results

### In silico evaluation of fungi-specific 18S rRNA gene sequence primer pairs

**Fig. 1:**
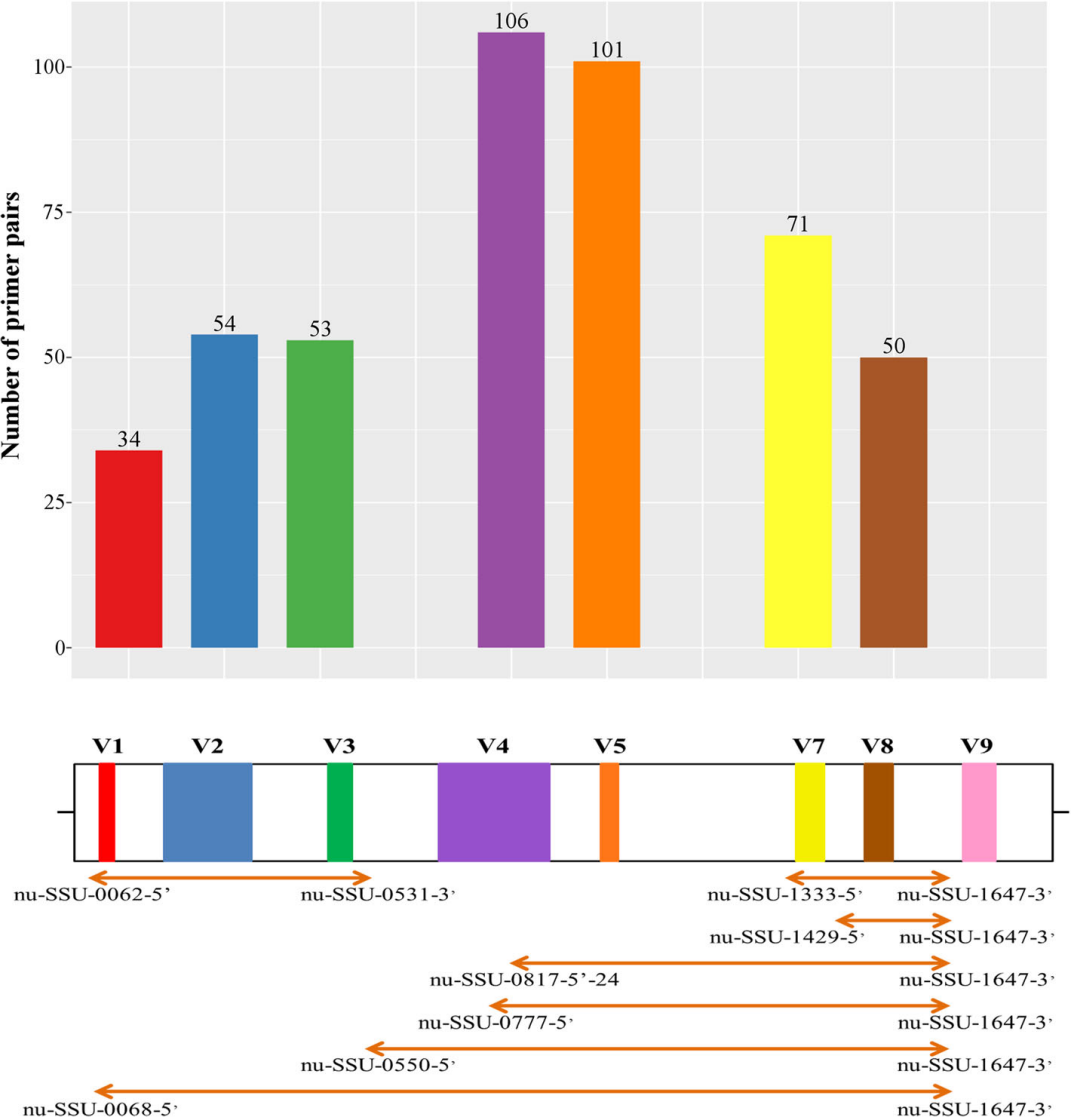
Primer pairs covering the different variable regions of the fungal 18S rRNA gene sequence. The fungal 18S rRNA gene sequence possess eight different variable regions, V1-V9 (V6 does not exist), colored differently. The barchart indicates the number of tested primer pairs covering a variable region with their amplicon. Amplicons produced by the seven top primer pairs as arrow lines. Primer names beside the arrow lines

The literature research revealed a total of 164 fungi-specific 18S rRNA gene sequence primers. 100 exhibited a fungal coverage rate of ≥50% with one mismatch. The highest fungal coverage rate observed among primers was 95.9% for zero and 98.1% for one mismatch, respectively (Additional file 1). Out of the 100 single primers, 436 different primer pairs were formed including pairs already proposed elsewhere (for example [11, 13, 14]). Amplicon products mainly spanned the V4 and V5 region of the 18S rRNA gene sequence targeting the other variable regions to a much less extent. None of the primer pairs matched the V9 region (Fig. 1). However, 89% of those primer pairs were excluded from further analyses as their overall fungal coverage rate fall below the acceptable threshold (Additional file 2).

A final number of seven primer pairs fulfilled the evaluation criteria. Three primer pairs were identified suitable for Illumina or Ion Torrent sequencing (*Group S)*, two for Sanger sequencing (*Group M)*, and two for third-generation techniques like PacBio (*Group L)*. None of them exceeded the value of 82.3 and 92.7% fungal coverage rate with zero and one mismatch, respectively (Table 1). All primer pairs targeted Dikarya sequences with a minimum of 70.5% under the condition of zero mismatches while the coverage rate of other phyla varied with the primer pair. However, group-specific coverage rates exceeded in general 70% under a one-mismatch-stringency with the exceptions of Cryptomycota, Entomophthoromycotina and Zoopagomycotina. Co-amplification of non-fungal eukaryotic sequences was low and ranged between 0.2–2.9% with zero mismatches. Thus, group-specific coverage rates of non-fungal eukaryotes stayed below 0.5% with some exceptions (Additional file 3).

**Table 1:** Characteristics and in silico performance of the best primer pairs. Primer pairs were grouped according to the expected amplicon size into three groups: S for small (≤600 bp), M for medium (600–1000 bp), and L for large size (> 1000 bp). Fungal and non-fungal eukaryotic sequence coverage rates tested by in silico PCR. Individual primer sequence and characteristics are listed in the Additional file 1. For primer pairs see Additional file 2

Primer pair	Old name	Amplicon (nt)	Variable regions covered	Fungi (%) (0 M/1 M)	Co-Amplif. (%) (0 M/1 M)
*Group S*					
nu-SSU-1333-5′/nu-SSU-1647-3′	FF390/FR-1	348	V7, V8	80.4/92.7	0.2/5.0
nu-SSU-1429-5′/nu-SSU-1647-3′	SR14R/FR-1	235	V8	76.8/86.0	0.8/2.5
nu-SSU-0062-5′/nu-SSU-0531-3′	TW9/GEO2	503	V1, V2, V3	73.7/89.1	1.5/8.0
*Group M*					
nu-SSU-0817-5′-24/nu-SSU-1647-3′	nu-SSU-0817-5′/FR-1	870	part of V4, V5, V6, V7, V8	75.8/86.2	0.5/4.5
nu-SSU-0777-5′/nu-SSU-1647-3′	Basid 3/FR-1	904	part of V4, V5, V6, V7, V8	68.3/80.8	2.9/14.7
*Group L*					
nu-SSU-0068-5′-20/nu-SSU-1647-3′	Fun18S1/FR-1	1615	all except V9	82.3/90.3	2.3/6.8
nu-SSU-0550-5′/nu-SSU-1647-3′	GEO3/FR-1	1133	V4, V5, V7, V8	73.1/88.4	0.9/2.0

*Amplicon (nt)* Length of generated amplicon

Fungi *(%*), coverage rate of fungal sequences with zero (0 M) and one (1 M) mismatch

*Co-Amplif. (%)* Non-fungal eukaryotic co-amplification rate under a zero (0M) and one (1M) mismatch stringency

### Amplification conditions and success of the proposed best primer pairs

The PCR conditions and the primer performance of the proposed seven best primer pairs were experimentally evaluated. The optimal annealing temperature for the different primer sets lay within the range of 42 to 45 °C (Additional file 4). Application of the primer-specific annealing temperature led to a successful amplification of the template DNA of 12 distant fungal taxa. The success was independent from the number of PCR replicates (Additional file 5).

### Design of fungi-specific primers generating a short amplicon (*Group S)*

The program ecoPrimers [15] suggested 20 candidate primer pairs, which were subjected to further in silico analysis with the TestPrime tool [16]. Only three primer pairs passed the evaluation criteria, all targeting the V4 and V5 region of the 18S rRNA gene sequence. Their overall fungal coverage rate ranged between 83.4 to 86.5% and 91.1 to 94.8% for zero and one mismatch, respectively. Fungal phyla and subphyla were homogenously targeted with a coverage rate of ≥70% under the condition of zero mismatches except Zoopagomycotina, Mucoromycotina and Entomopthoromycotina. Co-amplification of non-fungal eukaryotic sequences was high reaching 16.3 to 34.1% with zero mismatches. The highest co-amplification rate was reported for the genus *Telonema* being targeted with a minimum of 84.5% by the newly designed primers. Similarly, co-amplification caused by sequences of Stramenopiles and Alveolata exceeded for all primer pairs the coverage value of 50% (Additional file 6).

### Group-specific primer pairs

We have screened our dataset for primer pairs applicable for the classification of fungal isolates through Sanger sequencing. In total, 15 primer groups were defined, of which three showed high group specific coverage rate at the phylum level, namely for Blastocladiomycota, Cryptomycota and Chytridiomycota, and 12 at the subphylum level. The latter group included primers specific to the three ascomycete subphyla (Pezizomycotina, Saccharomycotina, Taphrinomycotina), three of the four basidiomycete subphyla (Agaricomycotina, Pucciniomycotina, Ustilagomycotina), all three mucoromycete subphyla (Glomeromycotina, Mortierellomycotina, Mucoromycotina), and the three zoopagomycete subphyla (Entomophthoromycotina, Kickxellomycotina, Zoopagomycotina). For most of the taxonomic groups, five promising primer pairs were identified and at least one of the primer pairs exhibited a group-specific coverage rate of 85% with zero mismatches. For Cryptomycota, Entomophthoromycotina, Kickxellomycotina and Zoopagomycotina only two, one, four and two primer pairs, respectively, were meeting the evaluation criteria matching sequences of the specific taxon group with a minimum of 70%. The majority of all primer pairs covered with their amplicons the V4 and V5 region of the 18S rRNA gene sequence. All relevant primer information can be found in the Additional file 7 including suggested annealing temperatures.

### Design of annealing blocking oligonucleotides

To reduce co-amplification, four different annealing blocking oligos were designed, targeting sequences of Stramenopiles, Alveolata, Rhizaria (SAR group), or *Telonema*, respectively. Blocking oligos are modified “primers” overlapping with the primer binding sites of co-amplifiable organisms and prevent elongation through a 3′-end modification. The designed oligos for Rhizaria and *Telonema* targeted the attachment site of the forward primer nu-SSU-1333-5´, while the oligos specific to Stramenopiles and Alveolata targeted the one of the reverse primer nu-SSU-1647-3´. A maximum of 77.1% target sequences were matched by the specific blocking oligos while the coverage rate for non-fungal eukaryotic sequences lay within the range of 0.2–18.7%. Overall fungal coverage rate was negligible with ≤0.1% and specific fungal groups were matched with a maximum of 0.8%. Solely, the Alveolata-specific blocking oligo covered 4.3% of the Zoopagomycotina sequences (Table 2, Additional file 8).

**Table 2:** Characteristics of the best blocking oligonucleotides complementing the primer pair nu-SSU-1333-5′/nu-SSU-1647-3′ (FF390/FR-1)

Target	Sequence	ComPrim	#nt	Tm (°C)	Fungi (%)	Alv. (%)	Rhiz. (%)	Stram. (%)	Tel. (%)
Alveolata	gtcgctcctaccgattga	nu-SSU-1647-3ˊ	16	50.3	0.08	52.6	6.3	0.9	3.3
Rhizaria	ttaacgaacgagacctcga	nu-SSU-1333-5ˊ	15	48.9	0	0	24.3	0.3	0
Stramenopiles	tcgcacctaccgattgaa	nu-SSU-1647-3ˊ	14	48.3	0	0.5	0.3	77.1	1.7
Telonema	gaccttaacctactaaatagtta	nu-SSU-1333-5ˊ	4	48.1	0	0.3	0	0	39.2

Fungal and non-fungal eukaryotic sequence coverage rate tested by in silico analysis

*ComPrim* Sequence complement to the indicated primer

*#nt* Number of identical nt’s shared by primer and blocking oligo sequence

*T*_*m*_ Annealing temperature

*%Fungi* Coverage rate for fungal sequences

*%Alv.* Coverage rate for Alveolata sequences

*%Rhiz.* Coverage rate for Rhizaria sequences

*%Stram.* Coverage rate for Stramenopiles sequences

*%Tel.* Coverage rate for Telonema sequences

### Fungal community survey with the primer pair nu-SSU-1333-5′/nu-SSU-1647-3′ (FF390/FR-1)

**Fig. 2:**
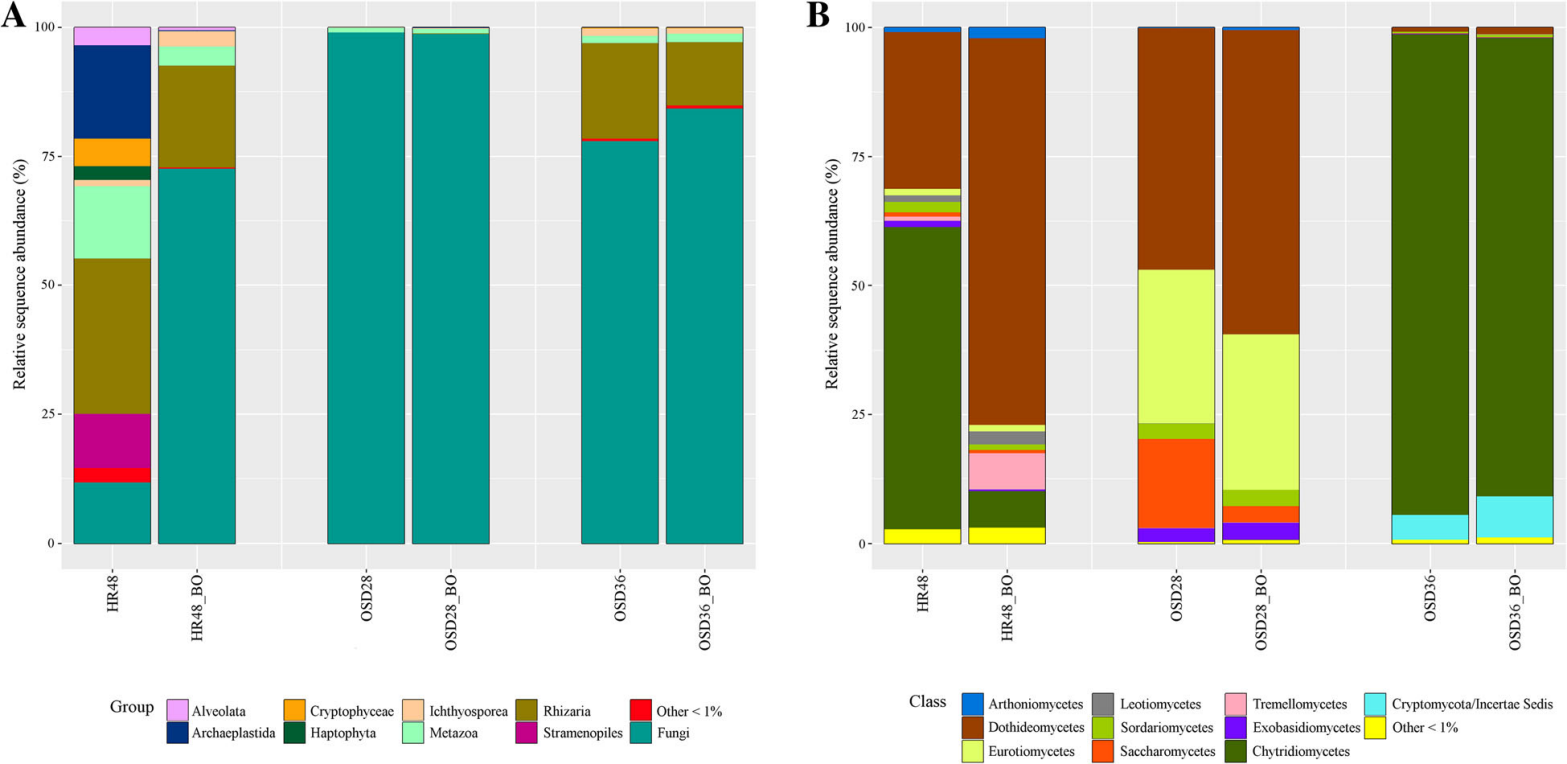
Taxonomic composition of three environmental samples split into fungal and co-amplified sequences. Barchart indicates relative sequence abundance of the different **a** eukaryotic groups, and **b** fungal classes amplified by the primer pair nu-SSU-1333-5′/nu-SSU-1647-3′ (FF390/FR-1) after rarefying sequences. Two libraries were prepared on the same template DNA: (i) solely with the primer pair, and (ii) primer pair and four different blocking oligonucleotides designed for Stramenopiles, Alveolata, Rhizaria and *Telonema* (BO). Others in **a**: Centrohelida, Choanoflagellida, Corallochytrea, Discicristoidea, Excavata, Freshwater Opisthokonta, Filasterea, Picozoa, Telonema. Others in **b**: Pezizomycotina *incertae sedis*, Lecanoromycetes, Lichinomycetes, Pezizomycetes, Agaricomycetes, Cystobasidiomycetes, Agaricostilbomycetes, Microbotryomycetes, Pucciniomycetes, Wallemiomycetes, Pucciniomycotina *incertae sedis*, Ustilaginomycetes, LKM15

A total of 1,840,354 sequence reads were generated for nine libraries (samples HR48, OSD28 and OSD36, with and without blocking oligonucleotides; three samples exclusively with blocking oligos) and assigned to 897,076 fungal sequences (Additional file 9). Amplification of the libraries with the primer pair nu-SSU-1333-5′/nu-SSU-1647-3’and no blocking oligos resulted in high co-amplification rates of non-fungal eukaryotic sequences. They made up to 88.2 and 22.1% of the relative sequence abundance in the samples HR48 and OSD36, respectively. Co-amplified products belonged mainly to the members of the Stramenopiles, Rhizaria and Alveolata. Amplification with blocking oligos increased fungal read abundance from 11.8 to 72.6% and from 77.9 to 84.3% in these two samples, while reducing the amount of non-target amplification (Fig. 2a). The fungal community of sample HR48 was dominated by Chytridiomycota (58.5%), Ascomycota (38.1%) and to a less extent by Basidiomycota and Cryptomycota, and showed a rich taxon composition on a lower taxonomic level. In contrast, the fungal community of sample OSD36 was mainly structured by taxa of the Chytridiomycota (93.1%) and to a very low extent by Ascomycota, Basidiomycota, Zoopagomycotina and Cryptomycota. In sample OSD28, Ascomycota was the dominating group with 97.1% of the relative sequence abundance and only few taxa of Basidiomycota and Chytridiomycota were detected (Fig. 2b).

UniFrac permutation tests revealed no significant effect of the blocking oligos on the fungal taxa composition (*p* > 0.05) when datasets were subsampled. For the non-subsampled dataset, only sample pair HR48/HR48_BO showed a significant difference for both Weighted and Unweighted UniFrac metrics (*p* < 0.05) (Additional file 10) being the sample with the highest reported co-amplification of 88%. Co-amplification rates up to 22% like for OSD36 did not affect the description of the fungal assemblages (Fig. 2b, Additional file 10).

### Comparison of the results obtained from a fungal and eukaryotic 18S tag sequencing and metagenomics approach

**Fig. 3:**
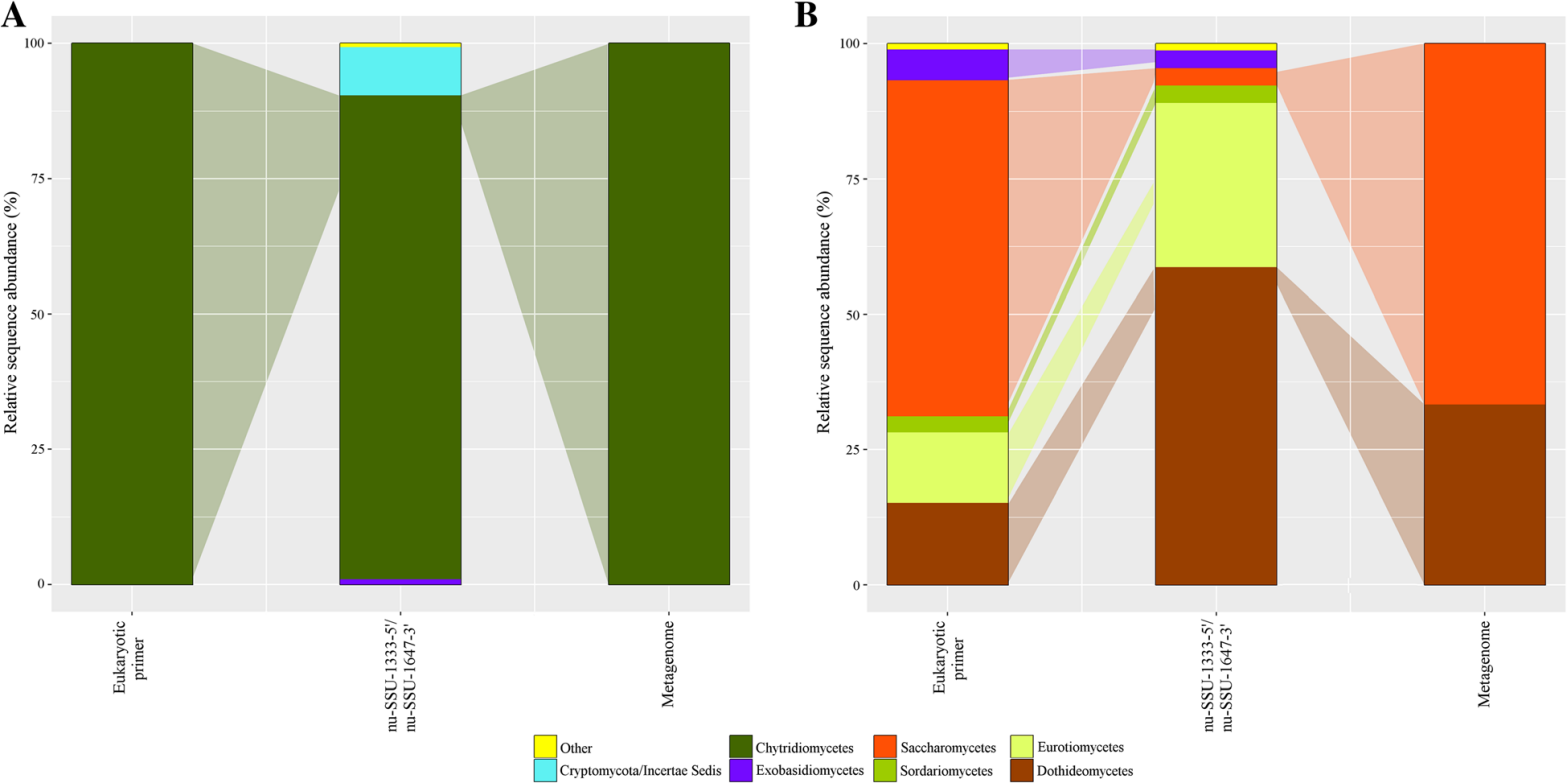
Comparison of three detection methods. Subsampled fungal communities were described by a eukaryote-specific primer (TAReuk454FWD1/TAReukREV3_modified), a fungi-specific primer (nu-SSU-1333-5′/nu-SSU-1647-3′ (FF390/FR-1)), and a metagenomics approach. Taxonomic composition as relative sequence abundance of sample **a** OSD36 and **b** OSD28. Colored shadows between bars show which proportion (approximated) the fungal sequences detected by the eukaryote-specific and metagenomics approach represent in the non-subsampled community of the fungi-specific approach. Others in **a**: Dothideomycetes, Sordariomycetes. Others in **b**: Eukaryote-specific approach: Agaricomycetes. Fungal approach: Arthoniomycetes, Lecanoromycetes, Pezizomycetes, Agaricomycetes

Within the Ocean Sampling Day (OSD) initiative 2014, the microbial communities of sample OSD28 and OSD36 were analyzed by a PCR-independent metagenomics and a general eukaryotic primer approach [17, 18]. The fungal sequence data thereby generated were compared to the fungal sequence dataset obtained with the primer set nu-SSU-1333-5′/nu-SSU-1647-3′ (FF390/FR-1). For OSD36, the fungal 18S tag sequencing detected all fungal taxa identified with the two other approaches being exclusively Chytridiomycetes. The fungal community amplified with the fungi-specific primers was composed of four additional classes. However, Chytridiomycetes clearly dominated the fungal 18S tag community with 90% of the relative sequence abundance matching the trend observed in metagenomics and eukaryotic 18S tag sequence data. Similarly for OSD28, the fungal primer approach detected all fungal taxa identified by the two other approaches. While the metagenomics dataset was exclusively composed of Dothideomycetes and Saccharomycetes sequences, common classes of the 18S tag datasets were Dothideomycetes, Eurotiomycetes, Sordariomycetes, Saccharomycetes, Exobasidiomycetes, and Agaricomycetes. Three additional classes were solely detected by the fungal specific primer approach. Saccharomycetes dominated communities of metagenomics and eukaryotic 18S tag sequencing while the fungal 18S tag sequencing dataset was dominated by Dothideomycetes sequences (Fig. 3).

### Primer performance on environmental samples

The fungal primer set nu-SSU-1333-5′/nu-SSU-1647-3’was further tested on samples from diverse habitat types, namely brackish water, freshwater, and marine sediment. Sequences were assigned to the five phyla of Ascomycota, Basidiomycota, Chytridiomycota, Cryptomycota and Mucoromycota and classified into 26 fungal classes/subgroups. The primer set captured the variations of the community structure over the three different habitat types. The brackish and freshwater samples were dominated by diverse clades of the Chytridiomycetes but clade-composition and abundance differed between these two sample types. Conversely the marine sediment sample was dominated by Dothideomycetes and Saccharomycetes, while Chytridiomycetes ranged on third position showing similar relative abundance values as Sordariomycetes and Cryptomycota. Seven fungal classes were uniquely identified in the marine sediment sample but all with relative sequence abundance below < 0.5% (Additional file 11).

## Discussion

### A list of seven top primer pairs for fungal community surveys

A final number of seven primer pairs were nominated to be the best performing one for fungal community surveys based on results of the in silico analysis (Additional files 2 and 3) and experimental testing (Additional file 5). However, none of the pairs exceeded the overall fungal coverage rate of 83% under a zero-mismatch-stringency (Table 1). The coverage rate is a crucial value for the power of PCR-based biodiversity assessments. Total universality is difficult to reach with a single primer pair especially for taxon rich kingdoms. Thus, similar coverage values were reported for proposed best primer pairs specific for bacteria, archaea [19] and eukaryotes [20]. The coverage rate depends on the discrimination power of the genetic region covered by the amplicon. For fungi, the variable regions V1, V4, V5, and V9 are the most discriminative ones [2]. Interestingly, the two best proposed primer pairs of the *Group S* covered the variable regions V7/V8 and V8 (Fig. 1) outperforming other primer pairs by targeting all major fungal groups and showing low co-amplification (Additional files 2 and 3).

Among the seven best primer pairs (Table 1) only primer pair nu-SSU-1333-5′/nu-SSU-1647-3′ (FF390/FR-1) was already introduced as primer combination [21, 22], while all others are newly composed pairs. In contrast, the combination of nu-SSU-0817-5′-24/nu-SSU-1200-3′ (old names: nu-SSU-0817-5′/nu-SSU-1196-3′) [11], currently one of the most prominent pairs used for fungal biodiversity assessments [23–25], failed to be included into the list of the top primer pairs as it did not target any sequence of the Entomophthoromycotina. Similarly, the primers nu-SSU-0214-5′ (EF4) and nu-SSU-1770-3′ (NS8) of the prominent combinations nu-SSU-0214-5′/nu-SSU-1729-3′ (EF4/EF3, [12]) and nu-SSU-0038-5′-19/nu-SSU-1770-3′ (NS1/NS8, [13]) were already discarded during the first evaluation step exhibiting too low overall fungal coverage rate (Additional file 1).

The in silico analysis revealed a taxonomic bias towards Dikarya for all the top primer pairs (Additional file 3). One problem is the overrepresentation of those groups in public sequence databases based on the fact that species of Dikarya form ~ 70% of the described fungal species [26] and Ascomycota is the species-richest fungal phylum [27]. Additionally, most of the fungi-specific 18S rRNA gene sequence primers were designed in the nineties or the beginning of the millennium (Additional file 1). Since then, sequence number of public databases constantly increased [28] including high numbers of sequences generated from environmental samples. The new sequence information led to the discovery of so far undiscovered fungal clades [29, 30] and the refinement of fungal taxonomy [31]. However, most existing 18S rRNA gene sequence primer pairs were not tested on updated sequence databases. Our results demonstrate that already a reshuffling of existing primers into new pairs increased their performance (Additional file 2) and underline the necessity of a regular reevaluation of existing primer sets.

Another step of our study was the design of primer pairs on a sequence database that included all recent submissions. Hereby, we focused solely on primer pairs generating a short amplicon (*Group S)* suitable for the sequencing techniques widely used for fungal community surveys. Several design strategies were tested leading to a final number of three primer pairs fulfilling the evaluation criteria. They exhibited an overall fungal coverage rate slightly higher than the ones identified by the literature research and covered the different fungal taxon groups more homogenously. In contrast, the non-fungal eukaryotic co-amplification of the three primer pairs was not acceptable and co-amplification targeted many more eukaryotic groups compared to the top primer pairs (Additional files 3 and 6). These results indicate the limitations of primer design for a relative conserved marker like the 18S rRNA gene aiming to target the great majority of sequences of a taxon-rich group as Fungi. When a certain threshold of fungal sequence coverage was reached, co-amplification rate quickly increased and vice versa. One possibility to overcome this limitation is the use of more than one primer set, which may, however, negatively affect comparability and semi-quantification.

Co-amplification is regularly reported for rRNA sequence-based fungal community surveys [7, 11, 12] and is caused by the conflict to find primer pairs possessing a low Shannon entropy [32] among fungal taxa but a high one against non-fungal eukaryotic groups. The position of the mismatch with non-target taxon groups is hereby very important as it influences the PCR sensitivity [33, 34]. Degenerated primers might be particularly prone to mismatching as the permuting position results in different binding energies to the nucleotides of the template DNA [35]. Three primer pairs of the top primer pair list have up to two wobbles. Surprisingly, their non-fungal eukaryotic co-amplification rate stayed below 3% with zero mismatches outperforming the newly designed primer pairs having no wobbles (Additional files 3 and 6).

### A compiled primer list for the amplification of fungal isolates by sanger sequencing

Fungi are a rich and promising source of novel biotechnological and medical agents. Compounds discovery often follows the classical discovery approach using culture based isolation techniques to screen isolates in bioassays [2]. The taxonomic classification of the isolated strains is based on a multiple-marker gene approach with the 18S rRNA gene sequence being one of the prominent markers [36]. Unfortunately, the 18S rRNA is reported to be the fungal marker with the highest PCR failure rate among markers of the rRNA [3]. The primer pair influences beside other parameters the PCR success. Thus, in case of amplification failure, a solution can be the change of the primer pair towards a pair with a high coverage rate for the target group. However, only few group-specific fungal 18S rRNA gene sequence primers have been designed [37–39] and in general, no information on coverage rates on lower taxonomic levels is provided for primers in literature. Thus, the selection of an appropriate alternative can be highly time-consuming.

In this study, we compiled an additional list with primer pairs independent of their overall fungal coverage rate but adequate for the amplification of diverse fungal phyla or subphyla. For each pair, amplicon length, variable regions covered, and a proposed annealing temperature was documented. As for the top primer list, the large majority of primer pairs were newly combined pairs (Additional file 7) outperforming existing pairs, which were specifically designed for a single taxon group [37].

### Performance of primer pair nu-SSU-1333-5′/nu-SSU-1647-3′ (FF390/FR-1) on environmental samples

Based on the in silico analysis, the primers nu-SSU-133-5′ and nu-SSU-1647-3′ (FF390 and FR-1) were proposed as best performing pair for the *Group S*. This primer pair is prominent for DGGE analysis [21, 40, 41] and less used for high-throughput sequencing [22, 42, 43]. In this study, we evaluated its performance with focus on taxonomic bias, co-amplification, and different source material. Fungal communities of four habitat types were analyzed. The results indicated a habitat-specific composition of the communities. Thus, Chytridiomycetes dominated nearly all aquatic fungal communities but differed in abundance and clade composition (Fig. 2, Additional file 11). Zoosporic fungi are known to significantly shape marine and freshwater communities being often highly abundant and playing important roles in the ecosystems [44–47]. In contrast, the fungal community of the sediment sample was more diverse being composed of 21 fungal classes (Additional file 11). Similar values have been reported for fungal soil communities [48, 49]. Additionally, many of the fungal classes detected with this primer set are among those one that dominate soil communities on a global scale [50]. These results attest for a good performance of the primer set nu-SSU-133-5′/nu-SSU-1647-3′, when applied to environmental samples independent from the habitat type, fungal diversity or composition. It further contrasts with the results of the in silico analysis, which showed a taxonomic bias towards Dikarya (Additional file 3). The primer set was able to capture the various fungal compositions even when the fungal community was dominated by non-Dikarya taxa (Fig. 2b, Additional file 11). This kind of discrepancy between results of in silico analyses and empirical tests is a well-known issue and emphasis the necessity to use both approaches to validate a primer pair for environmental studies. A careful selection of the primer set and the use of adequate PCR conditions further assist in receiving a reflection of the true picture of natural microbial communities [51].

In a final step, the results obtained for samples OSD28 and OSD36 through a fungal and eukaryotic 18S tag sequencing and PCR-free metagenomics approach were compared. In spite of certain statistical differences, the fungal primer approach detected all fungal taxon groups found by the two others but led to a deeper resolution of the fungal community, even when being subsampled (Fig. 3). Fungal sequences in marine metagenomics and eukaryotic 18S tag sequence datasets are generally represented by a small amount of the total sequence reads [52, 53]. Thus, for OSD28, only one OTU of the most abundant Dothideomycetes OTUs detected by the fungal primer approach was also detected by the eukaryotic 18S tag sequencing. Additionally, abundance values can significantly change when the same community is sequenced by different marker genes or (variable) regions [54]. Here, the V4 and V7/V8 of the 18S rRNA gene sequence was targeted by the eukaryotic and fungal 18S primer set, respectively. Metagenomics can recover a somehow similar taxonomic overview but suffer from the inability to infer fungal OTUs and from high uncertainty in identification [54] explaining the low fungal diversity detected in the two OSD samples (Fig. 3).

### Co-amplification of non-fungal eukaryotic sequences

Our results revealed that the matching of the primer sequence with the few co-amplified groups of the in silico PCR with 0.2 and 5% of relative sequence abundance under a zero and one mismatch-stringency, respectively, became a relevant problem in environmental surveys. Up to 88% of sequence reads of the marine samples were non-fungal co-amplified products (Fig. 2b). One possibility to reduce co-amplification of non-target organisms is the use of blocking oligos [55, 56]. In this study, four types of blocking oligos were designed targeting Stramenopiles, Alveolata, Rhizaria, and *Telonema* (Table 2).

The addition of these blocking oligos to the PCR and blocking of other non-fungal eukaryotic groups (Additional file 8) in the fungal community surveys resulted in a relevant reduction of the co-amplified sequences while none of the fungal groups were lost (Fig. 2b). Unifrac permutation tests confirmed no effect of the blocking oligos on the description of fungal assemblages (Additional file 10). Thus, the observed differences in abundance of fungal classes (Fig. 2b) were not caused by the presence of distant taxa in the two communities but by different abundance values of taxa being present in both assemblages. Anyhow, these differences were not significant. However, the high amount of co-amplified sequences biased the view on the fungal assemblage as shown for sample HR48 (Additional file 10), why the use of blocking oligos is recommended for samples risking high co-amplification. Nevertheless, the use of blocking oligos cannot guarantee a complete reduction of the target organisms (Fig. 2b). The SAR group consist of the most diverse protistan supergroups with more than 25,000 morphospecies of Stramenopiles and 10,000 of Alveolata and Rhizaria being described [8] and new clades being continuously discovered [57, 58]. The design of a single blocking oligo covering all sequences of such large and diverse target groups is not possible (Table 2). So far, only few studies were reporting the application of blocking oligos for environmental sequencing using up to two blocking oligos in the same PCR [59, 60]. We could show that the simultaneous use of four blocking oligos was effective. However, it is unclear if there is a limitation for the number of blocking oligos used in a single PCR, especially when “universal” blocking oligos are used which may co-effect each other and lead to uncontrolled co-blocking of sequences.

The primer pair nu-SSU-1333-5′/nu-SSU-1647-3′ (FF390/FR-1) has further been proposed as the candidate for quantifying fungal biomass by real-time Q-PCR [61]. The authors validate their results with a cloning/Sanger-sequencing step of fungal soil communities detecting no co-amplified products. They concluded that the primer pair is suitable for quantification of soil fungi and remark that non-fungal eukaryotic groups with a risk of co-amplification like Alveolata and Stramenopiles do not occur in soil. However, these groups display an abundant part of the diverse eukaryotic fractions in marine realms such as ocean surface water [53], deep sea and hydrothermal vents [62], and freshwater systems [63] and can reflect a significant portion of non-target amplification products (Fig. 2b) [7, 64]. Consequently, the application of the primer pair nu-SSU-1333-5′/nu-SSU-1647-3′ (FR-1/FF390) is not suitable for aquatic samples as realized by Taylor and Cunliffe [65] without a careful check of co-amplified groups by a sequencing step. Failure to do so can lead to sample amplicons being dominated by co-amplified non-fungal products leading to wrong fungal biomass estimations.

## Conclusions

The choice of primers is an essential step in the workflow of fungal taxonomic classification controlling the specificity of amplification. Most often, primer pairs are chosen based on comparable research studies, although they may not be the best choice in terms of efficiency and target specificity. This study revealed a high variation among 18S rRNA fungal specific primers and their characteristics, which reflects the variety of research issues and techniques for which, and the time point when, primers were designed. Thus, primer pairs highly differed in their (total) fungal coverage rate on higher as well as on lower taxonomic levels and in their non-fungal eukaryotic co-amplification. The total fungal coverage rate was for most of the primer pairs even too low to be recommended for the description of fungal communities. Only seven of the 439 tested primer combinations fulfilled the evaluation criteria. Surprisingly, six of them were new primer combinations of existing primers. Besides, some other primer pairs were identified as suitable candidates for the phylogenetic classification of isolates as they exhibit high coverage rates of specific fungal taxon groups. This illustrates the necessity for a careful selection of primer pairs and PCR strategies, which will differ dependent on the research question.

The in silico analysis attested that all primer pairs have very small rates of non-fungal eukaryotic co-amplification. These values are in the range of fungal primers in general, which are often neglected as they do not cause problems for the sequencing output. By contrast, co-amplifying groups were represented by high numbers of generated sequences in some samples of our study. For the primer pair nu-SSU-1333-5′/nu-SSU-1647-3′ (FF390/FR-1), this may be of special importance when applied to marine samples. Although our designed blocking oligos effectively reduced co-amplification, it may be necessary to adapt and/or design new blocking oligonucleotides for different type of sample and habitats. Most important, these results emphasize that “fungi-specific” 18S rRNA primers cannot directly be used for fungal biomass assessment by real-time Q-PCR without a prior assessment of the PCR specificity by a sequencing step.

The selection of the right primer pair adapted to the research issue and sequencing technique is often time-consuming. To remedy this issue, we developed this primer toolkit which provides the gap by providing in-depth information on fungal primers. The primer toolkit further complements the already existing (fungal specific) 18S rRNA gene sequence tools. In combination, they allow now an easy and straight-forward (phylogeny-based) classification of fungal query sequences in a user-friendly manner.

## Methods

### Compilation of a comprehensive primer list

A comprehensive literature research on fungi-(group)-specific 18S rRNA gene sequence primers was conducted in March 2015. Search engines like “Web of Knowledge” [66], “Google Scholar” [67] and “Google” [68] were browsed with keywords including “fungi”, “primer”, “SSU” or “18S”, “fungal community”, “environmental sample”, and names of fungal phyla/subphyla. If needed, the sequence format of the identified primers was adjusted to the IUPAC wobble system [69]. Primer-specific characteristics were calculated including the GC-content, basic and salt adjusted melting temperature (T_m_) using the program OligoCalc [70]. Positions of the primers were referenced to the 18S rRNA gene sequence of *Saccharomyces cerevisiae* (acc. No. Z75578, [71]). Finally primer naming was unified following the primer nomenclature system of Gargas & DePriest [72].

### In silico evaluations

The fungal coverage rate of all listed primers was tested by matching primers against the non-redundant SSU Ref SILVA database version r126 allowing zero or one mismatch using the TestProbe 3.0 tool [16]. The fungal coverage is defined as the percentage of fungal sequences from the total number of fungal sequences being matched by the primer. Only primers covering at least 50% of the fungal sequences with one mismatch were used for further analyses. Primers were assembled into pairs whenever the respective melting temperatures showed < 5 °C difference. The resulting primer pairs were divided into three groups according to the expected amplicon size: (i) *Group small (Group S)* with a generation of fragments ≤600 bp, (ii) *Group middle* (*Group M*) generation of fragments between 600 to 1.000 bp, and (iii) *Group large* (*Group L*) generation of fragments > 1.000 bp.

Primer pairs were subjected to in silico evaluations to analyze co-amplification, overall and fungal phyla/subphyla coverage rate using the same settings and sequence dataset as described above but using TestPrime 1.0 as evaluation tool [16]. Fungal taxonomy of the underlying SILVA dataset was manually adjusted to the new fungal taxonomy for zygomycete fungi [31]. Variable regions covered by the amplicon, amplicon length and start/end position was noted for each primer pair. For biodiversity assessments, only primer pairs meeting the following criteria were further shortlisted: (i) ≥ 65 and ≥ 75% fungal coverage with zero and one mismatch, respectively, (ii) targeting all major fungal phyla and subphyla, and (iii) < 20% co-amplification of non-fungal eukaryotic organisms with the parameter of one mismatch. Primer pairs were ranked based on the highest number of fungal coverage together with the lowest non-fungal eukaryotic co-amplification with special focus on groups reported to be highly problematic in marine samples [7, 64], namely Stramenopiles, Alveolata, Rhizaria, and *Telonema*.

To define best primer pairs suitable for classification of specific fungal phyla/subphyla, only primer pairs with < 20% co-amplification under a zero-mismatch-stringency and < 30% co-amplification under a one-mismatch-stringency were further analyzed. From those, up to five best primer pairs were recorded for each subphylum whenever the subphylum-specific coverage exceeded 70%. This search was solely conducted for the primer pairs belonging to the *Group M* as Sanger sequencing is the method of choice for classification of fungal isolates.

### Primer design for the amplicon category < 600 bp

It was further tested if a new primer pair for the *Group S* can be designed that outperforms the best primer pairs recognized by the above mentioned approach. Primer design was performed by the ecoPrimers program v 1.0 [15] using the manually curated high-quality 18S rRNA gene sequence alignment containing 12,870 fungal nearly full-length sequences [10]. To evaluate possible co-amplification, a non-fungal eukaryotic sequence reference database was prepared. Therefore, eukaryotic non-fungal sequences of the National Center for Biotechnology Information (NCBI) non-redundant nucleotide sequence Genbank database, release 213 [73] were used and enriched by sequences from the SILVA database being not redundant to the first one resulting into a final non-fungal eukaryotic 18S rRNA gene sequence number of 101,067. Different design strategies were tested changing parameters (0.5 < sensitivity quorum < 0.8; 0.5 < strict matching quorum < 0.8; 0.1 < false positive quorum < 0.3), target groups (all fungal groups; each fungal group separately), and databases (all fungi and outgroup sequences; only fungi; only basal fungi, i.e. excluding Dikarya and Glomeromycotina). The primer pairs were further filtered with the following parameters: (i) targeting all fungal groups, (ii) ≤ 20% co-amplification, (iii) ≤ 600 bp amplicon generation, (iv) primer length between 18 to 21, (v) most specific primer in the pair with the lowest Tm, and (vi) ≤ 10 °C Tm difference between both primers in the pair. Next, the 20 primer pairs with the highest minimum barcode coverage (*B*_*c*,_ the proportion of target species amplified *in-silico*)) and barcode specificity (*B*_*s*_, the proportion of species *in-silico* amplified which are unambiguously identified) value as well the lowest co-amplification rate were selected. Detailed match of the selected primers with the databases sequences were produced with ecoPCR program v 0.8 [74], allowing until 3 mismatches. When a primer matched the target groups with multiple variants, a consensus primer with degenerated nucleotides was built in order to improve the coverage of target groups. In a final step, consensus primer pairs were subjected to the same in silico evaluation approach described in the paragraph "In silico evaluations".

### Annealing blocking oligonucleotide design

Group-specific blocking oligonucleotides were designed for the eukaryotic SAR group and *Telonema* species targeting the annealing region of one of the two primers nu-SSU-1333-5′ and nu-SSU-1647-3′ (FF390/FR-1), [21, 22]) identified to form the best performing primer pair within the *Group S*. In a first step, the SILVA database was amplified with the best primer pair of the *Group S* using the ecoPCR program with the setting of a maximum of one mismatch per primer. Next, the in silico amplified sequences including the primer sequences at both ends were splitted among the different taxonomic groups and dereplicated. Dictionaries of 18- to 25-mer blocking oligos with at least 3 nt overlap with one of the two primers were created for each co-amplified outgroup. Finally, candidate blocking oligos were selected among those with the best coverage for the target group, the smaller cumulative coverage of fungal groups while having a similar T_m_ to the best fungi-specific primer pair of the *Group S*.

### Fungal cultures

As the selection of the best primer pairs was based on the in silico analysis, the next step was the proof of successful in vitro amplifications. Primer pairs were tested to amplify template DNA derived from various taxonomic fungal groups. Fungal taxa were selected to cover the major part of the fungal tree on higher taxonomic level. Thus, for each subphylum of the Dikarya and for three distant phyla of the Fungi *Incertae sedis* a representative taxon was chosen. Six of the 12 strains were obtained from the Deutsche Sammlung von Mikroorganismen und Zellkulturen (DSMZ) GmbH (Braunschweig, Germany), namely *Ustilago maydis* (DSM 4500, Ustilagomycotina, Basidiomycota), *Leucosporidium scottii* (DSM 4636, Pucciniomycotina, Basidiomycota), *Wallemia sebi* (DSM 5329, Wallemiomycotina, Basidiomycota), *Taphrina deformans* (DSM 4398, Taphrinomycotina, Ascomycota), *Coemansia erecta* (DSM 6933, Kickxellomycotina, Zoopagomycota), and *Allomyces arbuscula* (DSM 955, Blastocladiomycota). Two additional strains were isolated during an excursion to the Jadebusen (Germany, 53.441293, 8.295822) by Dr. Marlis Reich on the 11th of September 2013, namely *Davidiellaceae* sp. (CB2, Pezizomycotina, Ascomycota) and *Didymellaceae* sp. (CA1, Pezizomycotina, Ascomycota) from the seawater and surface sediment, respectively. Fungal strains were grown for 3 weeks at 18 °C in the dark on Malt Extract Peptone Agar (30 g/l malt extract, 3 g/l soya peptone, 15 g/l agar) (strain DSM 4500), Potato Dextrose Agar (20 g/l glucose, 15 g/l agar solved in infusion of potatoes) (strains DSM 4636, DSM 4398), YpSs Medium [75] (strain DSM 955), M 40 Y medium (400 g/l sucrose, 20 g/l malt extract, 5 g/l yeast extract, 20 g/l agar) (strain DSM 5329), or Czapek-Dox medium [76] (strains CB2, CA1). Six small colonized agar pieces were transferred to liquid medium, grown for 4 weeks at 18 °C in the dark on a Promax 2020 shaker (Heidolph, Karlsruhe, Germany) at 110 rpm. Biomass was harvested over a 3 μm particle retention round filter (Grade 389, Sartorius, Goettingen, Germany) and stored at − 20 °C for further treatment.

Cell material of four other fungal strains were provided by Prof. Dr. Imhoff from the KSMP (Kultur Sammlung Mariner Pilze) culture collection (GEOMAR, Kiel, Germany), namely *Candida mesenterica* (MF249, Saccharomycotina, Ascomycota), *Pichia anomala* (LF964, Saccharomycotina, Ascomycota) and *Mucor fragilis* (KF737, Mucoromycotina, Mucoromycota). A fruiting body of *Agaricus bisporus* (Agaricomycotina, Basidiomycota) was sampled from a compost heap in Bremen (Germany, 53.104635, 8.895263) by Dr. Marlis Reich on the 15th September 2015. It was cut under sterile conditions in pieces and the inner stem tissue directly below the carpophore was sampled and stored at − 20 °C.

Finally, the genomic DNA was extracted from 0.5 g of freeze-grinded tissue of each fungal species using the innuPREP Bacteria DNA kit (jenaAnalytica, Jena, Germany) following the manufacturer’s instructions.

### PCR efficiency of the best primer pairs for biodiversity assessments

In a first step, the optimal annealing temperatures for the best primer pairs of each amplicon-size group were defined in a gradient PCR approach: for each primer pair a range of eight different annealing temperatures was tested using the lower T_m_ of both primers within a pair as middle value. Subsequently, with steps of 0.5 °C three temperatures lower and four higher than the middle temperature were tested.

The PCR reactions were conducted in 20 μL volumes containing 1/10th volume of 10x Dream Taq DNA Buffer (Thermo Fischer Scientific, Darmstadt, Germany), 1 μM Bovine Serum Albumin (GeneON, Ludwigshafen, Germany), 200 μM dNTP’s (Fermentas Thermo Fischer Scientific, Pittsburgh, PA USA), 0.2 μM of each primer (Eurofins Genomics, Ebersberg, Germany), 0.5 U Dream Taq DNA polymerase (Thermo Fisher Scientific) and 50 ng/μL of the template DNA on a peqSTAR 2x double block thermocycler (peqlab Biotechnologie GmbH, Erlangen, Germany). Genomic DNA of *Taphrina deformans* and *Agaricus bisporus* served as template DNA. Each PCR was repeated three times independently.

The PCR conditions were as follows: initial denaturation at 94 °C for 4 min, followed by 30 cycles of denaturation at 94 °C for 30 s, calculated annealing temperatures for 60 s, extension at 72 °C for 90 s and a final extension step at 72 °C for 10 min. Successful amplification was checked on a 2% agarose gel, stained with ethidium bromide and visualized with a UVP Benchtop 2UV Transilluminator (UVP, LLC, Upland, USA). The best annealing temperature for a primer pair was defined as temperature where for both template strains the strongest band intensity was observed. In the case of having DNA bands of the same intensity, the median of the optimal temperature was chosen.

Finally, the in vitro performance of the four primer pairs was tested on the above mentioned 12 fungal species following the same PCR conditions and using the proposed best annealing temperature for each primer pair.

### Fungal community analysis

The best primer pair of the *Group S* (nu-SSU-1333-5′/nu-SSU-1647-3′) and its corresponding blocking oligos were tested for their performance in diverse fungal biodiversity assessments. Surface water biomass of three marine, one brackish water and one freshwater samples (all 0.2–0.5 m depth), as well as biomass of one sediment sample served as DNA templates. Two of the three marine samples were taken during the OSD campaign on the 21st of June 2014. Sample OSD28 originated from a back reef environment at Belize in the Caribbean Sea (16.8025, − 88.0816) and sample OSD36 from the Woodland Beach of Delaware, USA at the North Atlantic (39.3322, − 75.4699) (for more information see [18]). The third marine sample was taken at the HR station (54.1833, 7.9) on the 7th August 2015 kindly provided by Dr. Gunnar Gerdts and Dr. Antje Wichels of the Alfred-Wegener-Institute Helgoland (AWI). The brackish (53.9817, 8.405) and freshwater (53.4744, 9.9837) samples were obtained at two stations of a transect from the island of Helgoland to the Elbe river (Germany) during a cruise with the research vessel Uthörn on the 5th of August 2015. In all cases, a maximum of two liters of water was filtered on a Sterivex membrane (0.2 μm pore size, hydrophilic PVDF Durapore membrane, Merck, Darmstadt, Germany), stored at − 20 °C until DNA extraction with the Power Water DNA Isolation Kit (MoBio, Carlsbad, CA, USA) following the manufacturer’s instructions. The sediment sample was obtained from a 5 m long gravity core (HE443–010-3; 54.0865, 7.9701) which was collected on the RV HEINCKE, cruise HE443 on 30th of April 2015 kindly provided by Prof. Dr. Sabine Kasten. 5 g of sediment sample was collected at regular depth intervals and DNA was extracted from 1 g of sediments as described in Oni et al. [77].

For each of the three marine surface samples, two sequencing libraries were prepared: one solely with the fungi-specific primer pair and one including additional the four different group-specific blocking oligos. The libraries of the other three samples were all prepared with the addition of the blocking oligos. Library preparation and sequencing were performed at LGC Genomics GmbH, Berlin, Germany. All sequencing reactions were based upon an Illumina Miseq chemistry following the manufacturers’ instructions. Sequence data can be obtained from INSDC with accession number PRJEB25747.

Generated community data was compared to data obtained by two further approaches including a general eukaryotic primer based (TAReuk454FWD1/TAReukREV3_modified, [78]) and a PCR-independent metagenomics approach [17].

### Sequence processing analysis

Generated sequence reads were delivered in an already demultiplexed form from which adapter and primer sequences were removed. Further sequence processing followed the OSD’s protocol for 18S rRNA gene sequence data [17] including a merging, length and quality trimming step. Next, quality-checked sequence reads were clustered into operational taxonomic units (OTUs) and taxonomically assigned by the SILVAngs pipeline v 1.6 [16] based on the SILVA non-redundant database 123 using the default parameters but setting the sequence similarity threshold to 98%. OTUs represented by less than five sequence reads and/or no taxonomic assignment were discarded. Finally, sequences were subsampled using the sample with the smallest read output as a reference over the sub.sample function in Mothur v1.25.0 [79]. Sequence processing and assignment of the eukaryotic and metagenomics libraries followed the same conditions.

### Statistical analysis

To test for an effect of blocking oligos on fungal taxon groups, a UniFrac pairwise significance test was run [80]. For each of the samples HR48, OSD28, and OSD36, four datasets were compiled. They were composed of subsampled and non-subsampled community data generated solely with primers or primers and blocking oligos. The latter case aimed to test if the amount of co-amplified sequences affected the community structure. Tests were run with the program PyCogent 1.9 [81] using unweighted and weighted UniFrac metrics permuting 1000 times. Fungal assemblages of samples were defined to be significant different with a Bonferroni corrected *p*-value of *P* < 0.05.

## Additional files

Additional file 1: List of the 164 fungi-specific primers detected by a literature research. For each primer, performance, characteristics and source literature are provided. (XLSX 36 kb)

Additional file 2: List of the 436 fungi-specific primer pairs tested for their performance by in silico PCR. Primer pairs were grouped according to the expected amplicon size into three groups: *S* for small (≤600 bp), *M* for medium (600–1000 bp), and *L* for large size (> 1000 bp). (XLSX 124 kb)

Additional file 3: List of the seven most promising primer pairs for biodiversity assessments identified by in silico PCR. Primer pairs are suitable for different sequencing methods dependent on the expected amplicon size. Sequence coverage rate of diverse fungal and non-fungal eukaryotic groups as revealed by in silico PCR. (XLSX 15 kb)

Additional file 4: Annealing temperatures empirically evaluated for the most promising primer pairs. Two fungal strains, one of the Basidiomycota and one of the Ascomycota, served as template DNA. Intensity of the color indicates the strength of the amplification product detected by ethidium bromide staining. Red, template DNA from *Taphrina deformans*; Green, template DNA from *Agaricus bisporus*; *, optimal annealing temperature. (XLSX 60 kb)

Additional file 5: Performance of the most promising primer pairs empirically tested on 12 fungal strains. (XLSX 10 kb)

Additional file 6: List of the three newly designed primer pairs passing the evaluation criteria. Primer performance was tested as for other primer pairs. Characteristics and sequence coverage rates of fungal and non-fungal eukaryotic groups are given. (XLSX 12 kb)

Additional file 7: Primer pairs suitable for the amplification of specific fungal phyla/subphyla. Characteristics of the primer pair and sequence coverage rate of the target group is indicated. (XLSX 19 kb)

Additional file 8: List of the designed annealing blocking oligonucleotides for the eukaryotic groups Stramenopiles, Alveolata, Rhizaria and *Telonema*. Characteristics and sequence coverage rates of fungal and non-fungal eukaryotic groups are given. (XLSX 11 kb)

Additional file 9: Information on sequence generation and downstream analysis process. (XLSX 9 kb)

Additional file 10: UniFrac pairwise permutation test. The effect of blocking oligos (BO) and amount of co-amplified sequences on fungal community structure was tested. Bonferroni-corrected *p*-values are reported. Source datasets were the subsampled and non-subsampled community data of samples OSD28, OSD28_BO, OSD36, OSD36_BO, HR48, HR48_BO. *significant difference of fungal assemblage (*p* < 0.05). (XLSX 10 kb)

Additional file 11: Taxonomic composition of three environmental samples. Barchart indicates relative sequence abundance of the different fungal classes/subgroups amplified by the primer pair nu-SSU-1333-5′/nu-SSU-1647-3′ (FF390/FR-1). Others: Blastocladiomyetes, Glomeromycetes, Monoblepharidomycetes, Pucciniomycotina_Incertae sedis. (PDF 192 kb)

## Abbreviations

*Bc*: Barcode coverage
bp: Basepair
*Bs*: Barcode specificity
DGGE: Denaturing gradient gel electrophoresis
DNA: Deoxyribonucleic acid
dNTP: Deoxyribonucleotide triphosphate
DSM: Deutsche Sammlung von Mikroorganismen
DSMZ: Deutsche Sammlung von Mikroorganismen und Zellkulturen
HR: Helgoland Roads
INSDC: International nucleotide sequence database collaboration
ITS: Internal transcribed spacer
IUPAC: International union of pure and applied chemistry
KSMP: Kultursammlung mariner Pilze
M 40 Y: Medium for osmophilic fungi
min: Minute
NCBI: National center for biotechnology information
nt: Nucleotide
OSD: Ocean Sampling Day
OTU: Operational taxonomic unit
PCR: Polymerase chain reaction
PVDF: Polyvinylidene fluoride
Q-PCR: Quantitative polymerase chain reaction
RNA: Ribonucleic acid
rRNA: Ribosomal ribonucleic acid
S: Svedberg, unit for the sedimentation rate as for the 18S rRNA
SAR: Stramenopiles Alveolata Rhizaria
sec: second
SSU: Small subunit of RNA
T_m_: Melting temperature
V1-V9: Variable region 1–9 of the 18S rRNA gene sequence
YpSs: Yeast powder soluble starch
