# Correction to: A comprehensive fungi-specific 18S rRNA gene sequence primer toolkit suited for diverse research issues and sequencing platforms

**DOI:** 10.1186/s12866-019-1628-y

**Published:** 2019-11-08

**Authors:** Stefanos Banos, Guillaume Lentendu, Anna Kopf, Tesfaye Wubet, Frank Oliver Glöckner, Marlis Reich

**Affiliations:** 1 Molecular Ecology, Institute of Ecology, FB02, University of Bremen, Leobener Str. 2, 28359 Bremen, Germany; 2 Department of Soil Ecology, Helmholtz Centre for Environmental Research GmbH – UFZ, Halle-Saale, Germany; 3 Department of Ecology, University of Kaiserslautern, Kaiserslautern, Germany; 4 Microbial Genomics and Bioinformatics Research Group, Max Planck Institute for Marine Microbiology, Bremen, Germany; 5 Present Address: Department of Community Ecology, Helmholtz Centre for Environmental Research GmbH – UFZ, Halle-Saale, Germany; 6 Department of Life Sciences and Chemistry, Jacobs University Bremen gGmbH, Bremen, Germany; 7 German Centre for Integrative Biodiversity Research (iDiv) Halle-Jena-Leipzig, Leipzig, Germany

**Correction to: BMC Microbiol**

**https://doi.org/10.1186/s12866-018-1331-4**

Following publication of the original article [1], we have been notified that three of the primer names identified as most promising candidates for fungal community surveys were incorrectly renamed following the primer nomenclature system proposed by Gargas & DePriest [2]. Their positioning on the reference sequence had to be shifted 1 bp to the 3'-end. The same error occurred in some primer names listed in the additional files.

As consequence, the number of identical nucleotides shared by the most promising primers and the newly designed blocking oligo sequences increased in one (see Table 2).

In this correction, the revised supplementary materials are included.

## Supplementary information

**Additional file 1.** List of the 164 fungi-specific primers detected by a literature research. For each primer, performance, characteristics and source literature are provided.

**Additional file 2. **List of the 436 fungi-specific primer pairs tested for their performance by in silico PCR. Primer pairs were grouped according to the expected amplicon size into three groups: *S* for small (≤600 bp), *M* for medium (600–1000 bp), and *L* for large size (> 1000 bp).

**Additional file 3.** List of the seven most promising primer pairs for biodiversity assessments identified by in silico PCR. Primer pairs are suitable for different sequencing methods dependent on the expected amplicon size. Sequence coverage rate of diverse fungal and non-fungal eukaryotic groups as revealed by in silico PCR.

**Additional file 4. **Annealing temperatures empirically evaluated for the most promising primer pairs. Two fungal strains, one of the Basidiomycota and one of the Ascomycota, served as template DNA. Intensity of the color indicates the strength of the amplification product detected by ethidium bromide staining. Red, template DNA from *Taphrina deformans*; Green, template DNA from *Agaricus bisporus*; *, optimal annealing temperature.

**Additional file 5.** Performance of the most promising primer pairs empirically tested on 12 fungal strains.

**Additional file 7.** Primer pairs suitable for the amplification of specific fungal phyla/subphyla. Characteristics of the primer pair and sequence coverage rate of the target group is indicated.

**Additional file 8. **List of the designed annealing blocking oligonucleotides for the eukaryotic groups Stramenopiles, Alveolata, Rhizaria and *Telonema*. Characteristics and sequence coverage rates of fungal and non-fungal eukaryotic groups are given.

**Additional file 11.** Taxonomic composition of three environmental samples. Barchart indicates relative sequence abundance of the different fungal classes/subgroups amplified by the primer pair nu-SSU-1333-5′/nu-SSU-1647-3′ (FF390/FR-1). Others: Blastocladiomyetes, Glomeromycetes, Monoblepharidomycetes, Pucciniomycotina_Incertae sedis.
