# Supplementary material for: A comprehensive fungi-specific 18S rRNA gene sequence primer toolkit suited for diverse research issues and sequencing platforms
Source: BMC Microbiol. 2018 Nov 20;18:190. doi: 10.1186/s12866-018-1331-4 (PMC6247509; doi:10.1186/s12866-018-1331-4)

Additional file 11: Taxonomic composition of three environmental samples. Barchart indicates relative sequence abundance of the different fungal classes/subgroups amplified by the primer pair nu-SSU-1333-5'-a/nu-SSU-1647-3' (FF390/FR-1).

Others: Blastocladiomyetes, Glomeromycetes, Monoblepharidomycetes, Pucciniomycotina_Incerta sedis.

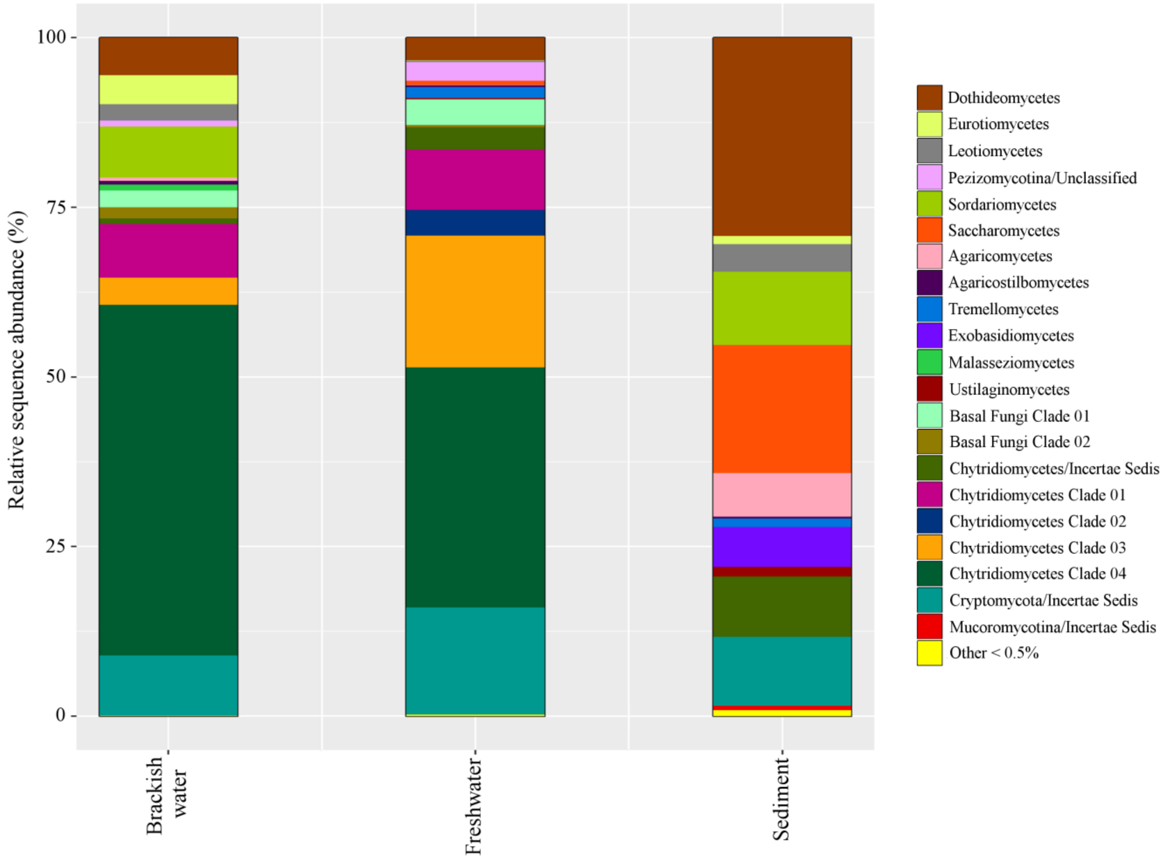

Supplement: Supplementary file 11 — Taxonomic composition of three environmental samples. Barchart indicates relative sequence abundance of the different fungal classes/subgroups amplified by the primer pair nu-SSU-1333-5′/nu-SSU-1647-3′ (FF390/FR-1). Others: Blastocladiomyetes, Glomeromycetes, Monoblepharidomycetes, Pucciniomycotina_Incertae sedis. (PDF 192 kb) [file 12866_2018_1331_MOESM11_ESM.pdf]
