# Supplementary material for: Correction to: A comprehensive fungi-specific 18S rRNA gene sequence primer toolkit suited for diverse research issues and sequencing platforms
Source: BMC Microbiol. 2019 Nov 8;19:249. doi: 10.1186/s12866-019-1628-y (PMC6842189; doi:10.1186/s12866-019-1628-y)

Additional file 11: Taxonomic composition of three environmental samples. Barchart indicates relative sequence abundance of the different fungal classes/subgroups amplified by the primer pair nu-SSU-1334-5'-a/nu-SSU-1648-3' (FF390/FR-1).

Others: Blastocladiomyetes, Glomeromycetes, Monoblepharidomycetes, Pucciniomycotina_Incerta sedis.

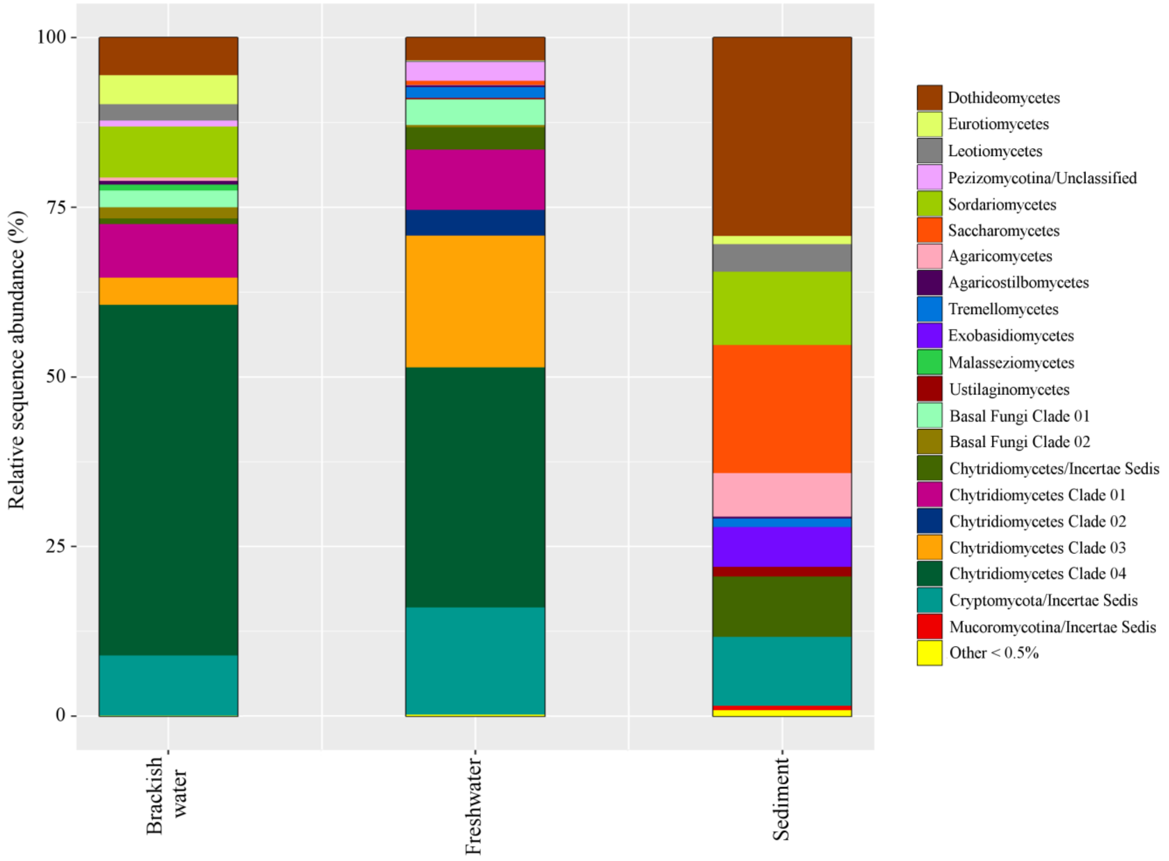

Supplement: Supplementary file 8 — Additional file 11. Taxonomic composition of three environmental samples. Barchart indicates relative sequence abundance of the different fungal classes/subgroups amplified by the primer pair nu-SSU-1333-5′/nu-SSU-1647-3′ (FF390/FR-1). Others: Blastocladiomyetes, Glomeromycetes, Monoblepharidomycetes, Pucciniomycotina_Incertae sedis. [file 12866_2019_1628_MOESM11_ESM.pdf]
